# Gemcitabine plus capecitabine (Gem–Cape) biweekly in chemorefractory metastatic colorectal cancer

**DOI:** 10.1007/s12094-014-1243-1

**Published:** 2014-11-27

**Authors:** P. Jiménez-Fonseca, M. P. Solis, M. Garrido, L. Faez, D. Rodriguez, A. L. Ruiz, M. L. Sanchez Lorenzo, E. Uriol, M. D. Menendez, J. M. Viéitez

**Affiliations:** 1 Medical Oncology Department, Asturias Central University Hospital, Carretera de Rubín s/n Finca “La Cadellada”, 33011 Oviedo, Asturias Spain; 2 Hemato-Oncology Department, Pontifical Catholic University, Santiago, Chile

**Keywords:** Refractory metastatic colorectal cancer, Gemcitabine, Capecitabine, Third-line chemotherapy, Progression-free survival, Overall survival

## Abstract

**Purpose:**

A proportion of patients with metastatic colorectal cancer (mCRC) are still able to continue with active therapy after their progression to fluoropyrimidines, oxaliplatin, and irinotecan regimens. Studies suggest that gemcitabine and fluoropyrimidines are synergic antimetabolites. The purpose was to evaluate gemcitabine–capecitabine (Gem–Cape) in heavily pretreated mCRC and to thus assess possible predictive factors for progression-free survival (PFS) and overall survival (OS).

**Patients and methods:**

This analysis was performed on 119 evaluable patients pretreated with fluoropyrimidines, oxaliplatin, irinotecan, and biological agents between June 2001 and July 2011. Patients received gemcitabine 1,000 mg/m^2^ day 1 and capecitabine 1,000 mg/m^2^ *bid* for 7 days every 2 weeks.

**Results:**

The general characteristics were ECOG 0–1, 89 %; male, 68 %, and median age 63 years. In total, 61 % had received two chemotherapy lines, while 39 % had received three or more. Objective response rates and stable disease rates at 3 months were 6.72 and 37.81 %, equalling a clinical benefit of 44.53 %. The median PFS and OS were 2.87 months [95 % confidence interval (CI) 2.53–3.17 months] and 6.53 months (95 % CI 5.33–8.77), respectively. The most frequent toxicities were grades 1–2, anemia (22 %), thrombocytopenia (10 %), and hand–foot syndrome (9 %); grade ≥3, diarrhea (2 %), with no treatment-related discontinuations. No treatment-related deaths were reported. Statistical significance was obtained by subgroups, assessing clinical benefits and objective responses for PFS and OS. Moreover, patients under 65 tended to have a better PFS.

**Conclusion:**

These data suggest that Gem–Cape is a tolerable and feasible regimen, associated with clinical benefit in non-selected, heavily pretreated, mCRC patients.

## Introduction

There is a lack of evidence on the use of chemotherapy in multi-treated, refractory metastatic colorectal cancer (mCRC) patients; moreover, alternative treatments have yet to be standardized their true benefit fully evaluated.

The published data of the few phase III studies [1, 2] that have been performed in this setting showed unsatisfactory results for overall survival (OS) with a median of 5.8–6.4 months, highlighting the need for further research and looking toward new active agents or new chemotherapy combinations.

Gemcitabine is a pyrimidine analogue that has been turned into difluorodeoxycytidine triphosphate (dFdCTP) and inhibits deoxyribonucleic acid (DNA) and ribonucleic acid (RNA) synthesis by four mechanisms: (1) inhibiting DNA polymerases (α, β, and δ); (2) adhering directly to DNA, thus prohibiting chain elongation; (3) inhibiting ribonucleotide reductase, resulting in decreased levels of essential deoxyribonucleotides for DNA synthesis; (4) and finally, incorporating itself into RNA, producing alterations in its processing and mRNA translation [3, 4].

**Fig. 1:**
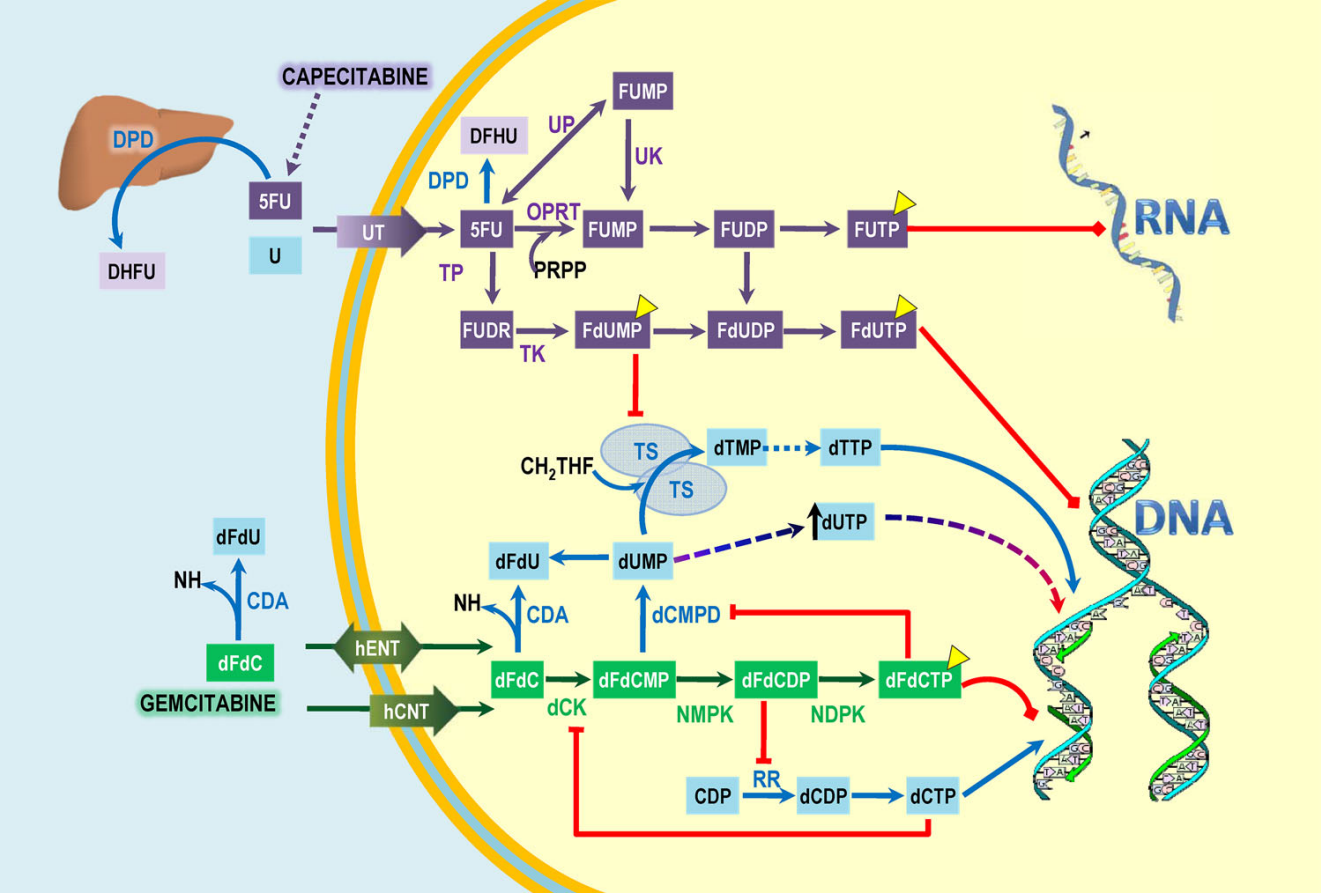
Interaction and synergism involving fluoropyrimidines and gemcitabine. *Yellow triangles* signal the active metabolites of this synergistic combination able to produce direct damage to nucleic acids. The *blue* pathway refers to normal integration of pyrimidines into DNA and RNA. The *purple line* represents fluoropyrimidines metabolism, just as the* green* pathway depicts to gemcitabine intervention. Crossroad and so synergism is located at tymidylate synthase (TS), being inhibited directly by fluoropyrimidine metabolite FdUDP and indirectly by the inhibition of the enzyme that catalyzes TS substratum, dUMP (deoxiuridine monophosphate)

Capecitabine, another antimetabolite, is an inactive prodrug that undergoes a complex process to reach its active form. Capecitabine conversion to 5-fluorouracil (5-FU) is higher in tumor cells than in normal tissues because of the higher expression of thymidine phosphorylase [5]. The depletion of the reduced nucleotide pool could increase the incorporation of the 5-FU metabolite fluorouridine monophosphate (FUTP) into RNA and of FdUTP into DNA, resulting in damaged RNA and DNA synthesis and function [6] (Fig. 1).

This analysis evaluates the survival, efficacy, and toxicity profile of gemcitabine–capecitabine (Gem–Cape) and intends to identify possible predictive factors in patients with mCRC treated in a single university hospital.

## Patients and methods

### Patient selection

Patients aged ≥18 years with a diagnosis of mCRC confirmed by biopsy and with documented evidence of failure of fluoropyrimidines; oxaliplatin; irinotecan; and biological agents, such as bevacizumab, cetuximab, or panitumumab, were treated with Gem–Cape.

All patients had measurable disease by RECIST criteria 1.1 [7], a performance status of the Eastern Cooperative Oncology Group (ECOG PS) from 0 to 2 [8], adequate bone marrow reserve (absolute neutrophils ≥1,500/µl and platelets ≥100,000/µl), adequate hepatic function (total serum bilirubin ≤3, the institutional upper limit of normal (ULN), phosphatase alkaline ≤10 institutional ULN, and AST ≤2.5 institutional ULN), and normal renal function (serum creatinine <1.5 mg/dl or creatinine clearance >60 ml/min).

Patients could have received first and subsequent lines of chemotherapy within a clinical trial, but this treatment must have been stopped at least 4 weeks in advance; radiotherapy or major surgery was allowed if it had been completed at least 6 weeks before starting Gem–Cape treatment. All patients signed written informed consent, and the compassionate use of gemcitabine was approved by the local ethics committee.

### Treatment

The Gem–Cape regimen used throughout the entire period consisted of gemcitabine 1,000 mg/m^2^ IV for 30 min on day 1 and oral capecitabine 1,000 mg/m^2^/12 h on days 1 through 7, repeated every 2 weeks.

Treatment fulfillment was evaluated by the oncological nurses’ registry in the case of gemcitabine and by the patient’s daily dietary notes in the case of capecitabine.

Patients were treated until they reached progressive disease (PD) on computed tomography scan (CT), unacceptable toxicity, withdrawal of patient consent, patient refusal, or at the investigator’s discretion.

Toxicity was assessed using the Common Toxicity Criteria (CTC-NCI) version 3.0 (http://ctep.cancer.gov) [9], and doses were titrated based on the laboratory values and tolerance. Treatment was withheld until resolution of grade 3 or 4 hematological toxicities or of non-hematological toxicities grade ≥2, except for alopecia and nausea. According to standard clinical practice, gemcitabine and capecitabine doses were reduced by 20 % if grades 3–4 neutropenia was present. Likewise, capecitabine alone was reduced by 20 % if grade ≥2 hand–foot syndrome of mucositis or grades 3–4 diarrhea was present on day 15 of any cycle.

### Pretreatment assessments

Pretreatment evaluations included complete physical examination, ECOG PS, weight, complete blood count, biochemistry profile (hepatic and renal function tests), carcinoembryonic antigen (CEA), and a baseline thoraco–abdomino–pelvic CT. Bone scans, magnetic resonance imaging, and ultrasound endoscopy were carried out only if clinically indicated. Physical examination, PS, weight, evaluation of adverse events, and the laboratory studies listed above, including CEA, were practiced before every cycle.

Tumor assessment according to the RECIST 1.1 criteria was carried out every 4 cycles (2 months). In addition, after PD or withdrawal from the study medication, the patients were followed monthly until death.

### Study end points

Primary end points assessed activity, evaluated as objective response rates (ORR) and progression-free survival (PFS). Secondary end points were OS, toxicity profile, and the possible predictive factors (age, PS, number of metastatic sites, surgery, previous monoclonal antibodies use, K-ras status, ORR, and clinical benefit) that might influence PFS and OS.

### Statistical analyses

The retrospective analysis of prospective recorded data included efficacy analyses of all patients with an intent-to-treat (ITT) principle. Safety analyses included all patients who received at least one dose of Gem–Cape.

The complete response (CR) and partial response (PR) were considered ORR, and clinical benefit was defined as ORR plus stable disease (SD), as measured by CT.

PFS was defined as the time between the first date of treatment administration until the first documentation of progression and last date of follow-up or death. Treatment-related death was defined as any death occurring within 30 days following the last dose of this regimen. For patients without disease progression at the time of the final analysis, the data on PFS were censored at the last assessment of the tumor status or at the discontinuation of treatment due to toxicity.

Likewise, OS was defined as the time interval between the first date of treatment administration and the day of death from any cause or the last known alive date (patients who were alive at the time of the analysis were censored for survival at the time of their last contact).

Descriptive statistics were reported as proportions and medians, and all analyses were performed with the log-rank test for one-sided *p* values, with an alpha value of 0.05. Meanwhile, the Kaplan–Meier method was used to estimate PFS and OS. The hazard ratio (HR) was calculated by the Cox regression function as median values and 95 % confidence intervals (CI).

The univariate and multivariate analyses of subgroups included the following: age (<65 vs. ≥65 years), sex (male vs. female), ECOG PS (0–1 vs. 2), location of the primary tumor (colon, rectum–sigmoid vs. rectum), primary reason for surgery (none, curative, or palliative), tumor burden (1–2 vs. ≥2 metastatic locations), ORR (CR and PR vs. SD and PD), clinical benefit (yes vs. no), previous biological therapy (yes vs. no), K-ras mutational status (wild type, mutant vs. unknown), previous chemotherapy sequence (oxaliplatin–irinotecan/irinotecan–oxaliplatin), and Gem–Cape line of treatment (third vs. further). We used Pearson’s Chi square test for qualitative variables and Student’s *t* test and ANOVA for quantitative variables. Logistic regression and Cox proportional hazards models were the multivariate models applied to identify the effects of the predictive factors listed above on PFS and OS. Statistical analyses were performed using the software R version R 2.15.3 under the terms of the Free Software Foundation’s GNU General Public License in source code form. Each regression coefficient (in terms of interaction) was estimated according to the maximum likelihood method and then statistically tested against the null hypothesis.

## Results

### Patient population

A total of 119 patients (ITT population) were treated between June 2001 and July 2011 (see baseline characteristics in Table 1).

**Table 1:** Characteristics of patients with refractory mCRC treated with Gem–Cape regimen

	*n* = 119	Percent
*Age*		
<65 years old	68	57.1
≥65 years old	51	42.9
*Sex*		
Females	37	31.1
Males	82	68.9
*ECOG*		
0–1	59	49.6
2	60	50.4
*Primary tumor localization*		
Colon	51	42.9
Rectum–sigmoid junction	68	34.1
Rectum		23
*Primary tumor surgery*		
None	9	7.6
Curative	51	42.9
Palliative	59	49.6
*Tumor burden*		
1–2 metastatic localizations	62	52.1
>2 metastatic localizations	57	47.9
K-ras status	47	39.5
Wild type	21	44.7
Mutated	26	55.3
*Previous use of monoclonal antibodies*		
No	62	52.1
Yes	57	47.9
Previous chemotherapy sequence		
Oxaliplatin–irinotecan	72	60.5
Irinotecan–oxaliplatin	47	39.5
*Gem–Cape line of treatment*		
Third	72	60.5
Fourth	47	39.5

The proportion of males was 68.9 %; median age was 63 years (range 36–79), and 89 % had an ECOG PS of 0 or 1. The location of the primary tumor was the colon in 43 %, rectum–sigmoid junction in 34 %, and the rectum in 23 %. The most common metastatic sites were liver (80 %), lymph nodes (58 %), lung (53 %), peritoneum (28 %), and bone (12 %). In first-line therapies, 8.4 % (*n* = 10) of patients received fluoropyrimidines in monotherapy; 66 % (*n* = 79) received fluoropyrimidine + irinotecan ± monoclonal antibodies; and 25 % (*n* = 30) received fluoropyrimidine and oxaliplatin ± biological agents. Capecitabine was the preferred fluoropyrimidine, used in 53 % of first-line and 70 % of second-line therapies. Monoclonal antibodies were administered in 34 % of first-line (*n* = 40) and in 24 % of second-line therapies (*n* = 29). Gem–Cape was used in third line in 61 % of the patients, and 39 % of cases received this therapy in successive lines.

### Efficacy

The median exposure of patients to Gem–Cape treatment was 3.23 months (range 0.43–47.2) and 6 cycles (range 1–23).

One case of CR (0.84 %), seven PR (5.88 %), 45 cases of SD (37.81 %), and 66 cases of PD (55 %) were observed. The ORR by ITT analysis was 6.72 %. The patient who reached CR underwent hepatic metastasectomy with complete morphological response, and 4 years after surgery, the patient maintains a recurrence-free disease.

**Fig. 2:**
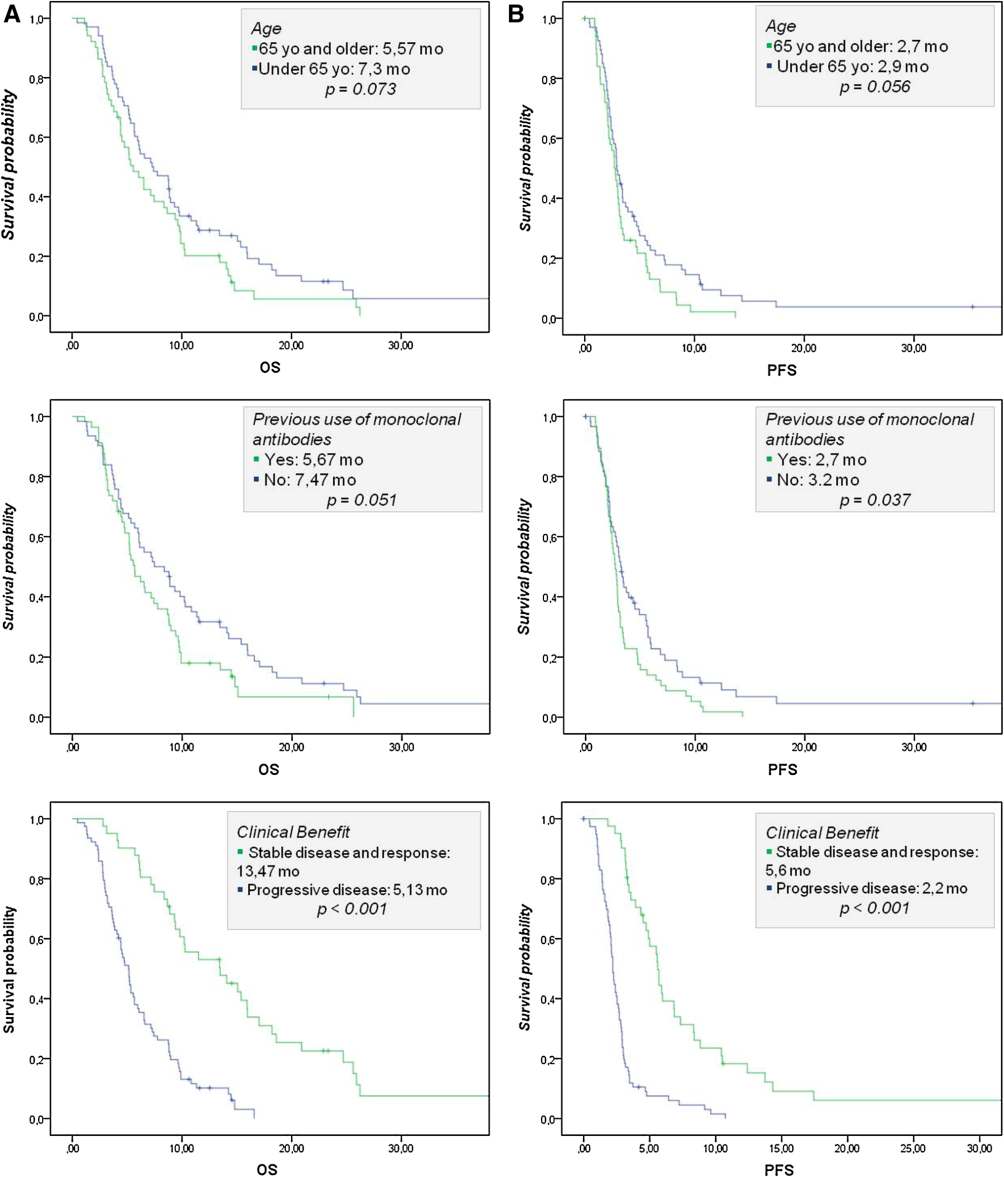

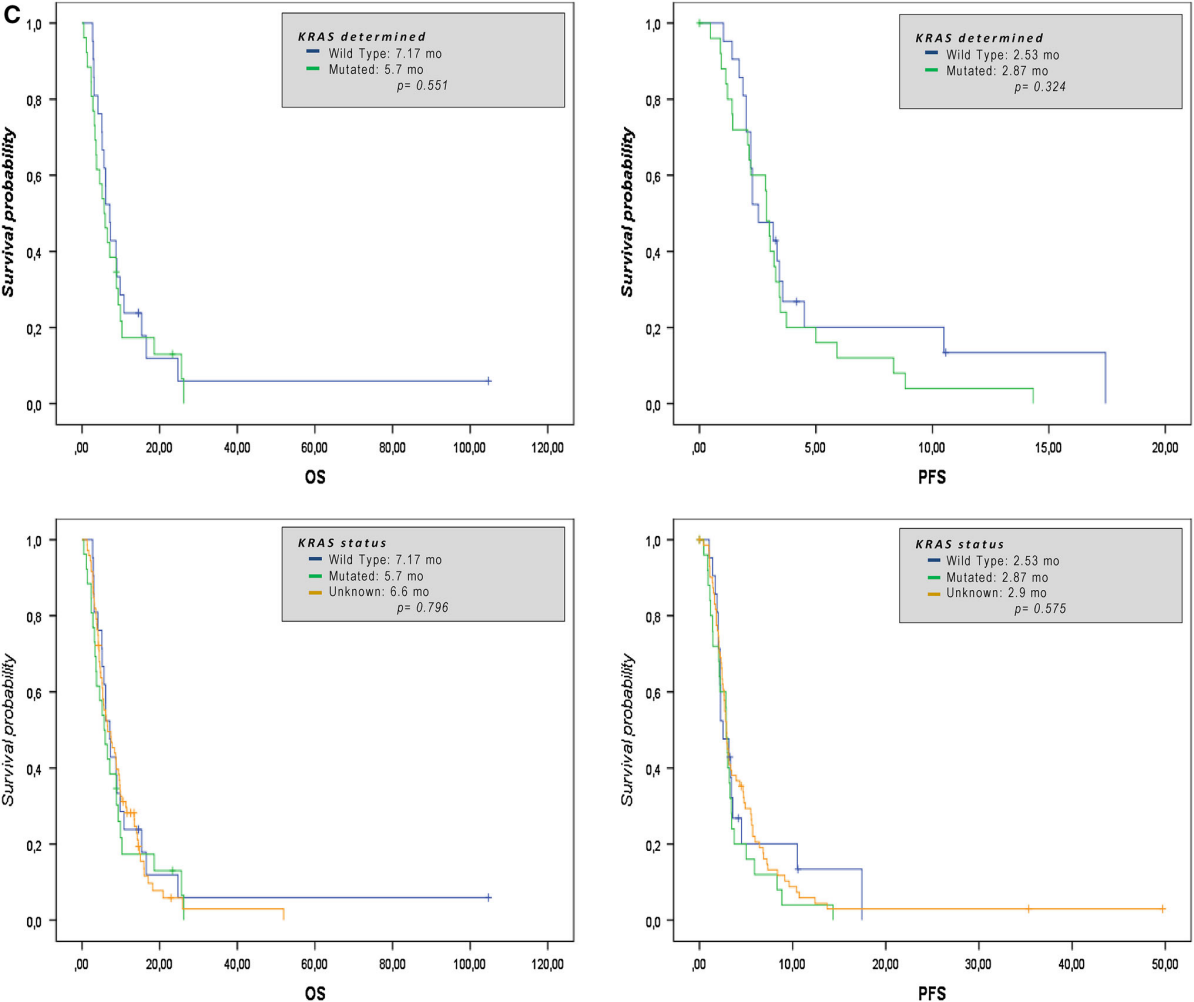
Kaplan–Meier survival curves for OS (**a**, *left panel*), PFS (**b**, *right panel*) and according to K-ras mutation status (**c**)

The median PFS was 2.87 months (95 % CI 2.53–3.17) and the median OS was 6.53 months (95 % CI 5.33–8.77). The survival rate was 50 % at 6 months and 25 % at the first year. Figure 2a–b shows the Kaplan–Meier estimation for PFS (A) and OS (B).

Univariate analysis showed statistically significant differences for PFS in patients who reached ORR (HR 0.147; 95 % CI 0.046–0.476; *p* = 0.001) or a clinical benefit (HR 0.242; 95 % CI 0.156–0.374; *p* = 0.0001). Statistical significance was observed for the association between OS and the previous use of monoclonal antibodies in favor of those who had not received them. Furthermore, both ORR and clinical benefit were the most significant factors affecting survival and PFS in univariate analysis. Younger patients (<65 years) showed a trend toward better survival (*p* = 0.056).

A Cox proportional hazard model was used to adjust survival curves, taking into account other factors that might influence PFS or OS. In the multivariate analysis, significant differences were found in favor of the subgroups with lower tumor burdens and those who achieved clinical benefit and response. For clinical benefit, the logistic regression analysis revealed lower survival rates in those who had previously used biological agents. Given that K-ras status determination only started to come on line as standard practice in 2008, this datum was available for only a limited number of patients. Despite this, the examination of the results according to “Unknown,” “Mutated,” and “Native” K-ras status did not yield statistically significant differences (Fig. 2c).

The previous sequence of cytotoxic agents did not represent significant differences in survival or clinical benefit (Table 2).

**Table 2:** Multivariate analysis for PFS, OS, and clinical benefit

	Progression-free survival (cox regression)				Overall survival (cox regression)				Clinical benefit (logistic regression)			
	*p*	HR	CI 95 %		*p*	HR	CI 95 %		*p*	HR	CI 95 %	
			INF	SUP			INF	SUP			INF	SUP
Age <65	0.108	1.404	0.928	2.125	0.252	1.290	0.834	1.996	0.748	0.870	0.371	2.038
KPS <70	0.490	1.152	0.771	1.721	0.498	0.864	0.567	1.318	0.204	0.583	0.253	1.341
Tumor burden	0.424	1.174	0.792	1.739	0.739	0.936	0.634	1.381	0.480	0.743	0.326	1.694
Previous McAb	0.139	0.729	0.480	1.108	0.402	0.835	0.548	1.272	**0.001**	**0.246**	**0.105**	**0.575**
Previous chemotherapy Sequence	0.758	1.074	0.683	1.687	0.071	1.529	0.965	2.422	0.589	1.289	0.513	3.240
Line	**0.006**	**1.803**	**1.187**	**2.739**	0.052	1.506	0.997	2.275	0.835	1.095	0.467	2.569
ORR achieved	**0.007**	**0.181**	**0.052**	**0.632**	0.086	0.339	0.099	1.166				
Clinical benefit	**0.000**	**0.223**	**0.136**	**0.367**	**0.000**	**0.235**	**0.138**	**0.401**				

Bold values indicate statistical significance (*p* < 0.05)

*KPS* Karnofsky performance status, *McAb* monoclonal antibody, *ORR* overall response rate, *HR* hazard ratio, *CI* confidence interval, *INF* inferior, *SUP* superior

### Toxicity

The toxicity events are listed in Table 3, with 94 % experiencing the most frequent grade 1–2 events: hand–foot syndrome (66 %), anemia (23 %), and vomiting (11 %). Grades 3–4 toxicity occurred in 6 % of the episodes, most of which were diarrhea. No treatment-related deaths were documented.

**Table 3:** Toxicity of Gem–Cape according to NCI CTC version 3.0 criteria

Toxicity	Grades 1–2		Grades 3–4	
	*n*	%	*n*	%
Anemia	26	22.61		
Thrombocytopenia	12	10.43		
Hand–foot syndrome	10	8.70	1	0.87
Asthenia	10	8.70		
Nausea/vomiting	9	7.83	1	0.87
Pseudoflu syndrome	7	6.09		
Mucositis	7	6.09		
Diarrhea	7	6.09	3	2.61
Neuropathy	3	2.61		
Alopecia	3	2.61		
Allergic reactions	1	0.87		

Treatment compliance was 100 %. Delays were necessary in 13 % of cases (15 patients), and dose reduction was required in 6 % of cases (7 patients).

## Discussion

The use of irinotecan, oxaliplatin, fluoropyrimidines, and the incorporation of biological agents such as bevacizumab, cetuximab, and panitumumab, has improved considerably ORR, PFS, and OS in patients with mCRC [2, 10–12]. Nevertheless, despite all of these drugs, patients become refractory; their lack of any standardized treatment alternatives reflects the necessity to continue efforts to uncover new schedules or drugs that are not only beneficial in terms of their action, but also in terms of their tolerability and effects on the quality of life.

Currently, very few trials have clearly shown a survival advantage for any regimen or particular drug in chemorefractory patients with mCRC; instead, the main advance has been the incorporation of antitarget therapies, such as panitumumab and regorafenib, both of which have been evaluated in phase III studies [2, 12]. Regorafenib showed an ORR of 1 %, disease control in 41 %, PFS of 1.9 months, and OS of 6.4 months. In comparison with placebo, regorafenib produced a benefit of 1.4 months, but also grade 3 toxicity (hand–foot syndrome, fatigue, hypertension, diarrhea, and rash) in more than 5 %. With conventional chemotherapy, nevertheless, advances have been limited. Thus, Gem–Cape has emerged as an exploratory treatment due to the synergy of these antimetabolites [13].

In vitro studies in CRC have demonstrated the synergy of gemcitabine with 5-FU [14], and phase I and phase II trials have evaluated doses, efficacy, and toxicity of this combination in patients with refractory tumors, especially in those with pancreatic and colorectal cancers [15]. Thus, gemcitabine and fluoropyrimidines have been evaluated in at least eight phase I or phase II studies [13, 15–21], showing an ORR of 30–38 % in naive patients but of 3.8–10.8 % in refractory cases after the failure of two or more chemotherapy lines during oxaliplatin and irinotecan regimens, with PFS of 2.7–4.2 months, and OS of between 8.9 and 11.3 months. Toxicity in these studies was manageable [22]. These data suggest that Gem–Cape is very attractive in patients with refractory mCRC, as demonstrated in some preclinical and phase I–II trials [13, 15–21]. Still, questions as to appropriate dosages and schedules have not been fully resolved. In the present series, most patients used capecitabine in second line; maintenance of activity and synergy with other drugs, including gemcitabine, was sought in third and subsequent lines of treatment. In order to take advantage of this synergy and consolidated activity of fluoropyrimidines in mCRC, we used a combination of Gem–Cape after the disease had progressed to oxaliplatin- and irinotecan-based regimens.

No serious toxicity was detected, and all patients completed treatment, with 13 % requiring cycle delays and 6 % dose reductions. Our series appeared to exhibit less toxicity than Bitossi’s report in 2008, who used continuous infusion of 5-FU with 36.1 % discontinuation or dose reduction rate that increased to 69.4 % in cases of gemcitabine [13]. The same combination was chosen for pancreatic cancer, which was treated with gemcitabine on days 1, 8, and 15 and with capecitabine for 14 days every 3 weeks, yielding grades 3–4 neutropenia in 22 % of the patients [23]. In this series, using gemcitabine on day 1 and weekly capecitabine for every 2-week cycle, the rate of grades 3–4 neutropenia was 0 %. Therefore, Gem–Cape is more manageable and tolerable than a continuous infusion of 5-FU associated with gemcitabine weekly for 3 weeks.

Despite the lower-dose intensity, the ORR achieved was 6.72 %; disease control 44.53 %; PFS 2.87; and OS 6.53 months. It was probably influenced by second line and no monoclonal treatment in a subgroup of patients. These data are comparable to those reported in heavily pretreated mCRC [13, 17, 18]. The ORR and clinical benefit were relevant because they are seemed to be the best predictive factors for survival (OS and PFS) in multivariate analysis. Bitossi et al. [13] published an ORR of 10.8 % in 37 patients, Fillos et al. [17] reported 3.8 % in 26 patients, and Qiu 0 % in 12 patients [18]. In 2009, Merl et al. [22] published a review of refractory mCRC patients with a PFS of 2.7–4.2 months. Thus, based on the historical data, this regimen appears to provide a clinical advantage over combinations using a gemcitabine dose intensity of 1,000 mg/m^2^ and capecitabine dose intensity of 2,000 mg/m^2^, 7 days every 2 weeks; this combination has been shown to have a similar ORR to that of others reported schemes, as shown in Table 4.

**Table 4:** Studies with gemcitabine and fluoropyrimidines in refractory mCRC

Study	Chemotherapy	Dose mg/m^2^/d	Frequency	N	RR (%)	Disease control (%)	mPFS	mOS	Neutrop. G3/4 (%)
Fillos [17]	Gemcitabine	750	Day 1 every 7 days × 6	26	3.8	34.8	2.7	11.3	45
	5-FU	450	Day 1 every 7 days × 6						
Pachon [19]	Gemcitabine	800–1,250	Day 1, 15 every 21	18	0	70	3.7	9.9	0
	5-FU (IC)	200	Every 21 days						
Bitossi [13]	Gemcitabine	1,000	Day 1, 8, 15 every 28	37	10.8	61.8	4.2	8.9	8
	5-FU (IC)	200	Every 28 days						
Madaje Wicz [20]	Gemcitabine	900	Day 1 every 7 day × 6	21	38	NR	NR	NR	11
	5-FU	450	Day 1 every 7 days × 6						
Schilsky [15]	Gemcitabine	1,000	Day 1, 8, 15 every 28	21	9.6	14.4	NR	NR	7.5
	Capecitabine	1,660	21 day every 28						
Fernandez [21]	Gemcitabine	900	Day 1 cada 14	21	NR	NR	4	9	NR
	Capecitabine	2,500	7 day every 14						
Qiu [18]	Gemcitabine	1,000	Day 1, 8 cada 21	12	0	36.4	2.27	5.57	17
	Capecitabine	2,000	14 day cada 21						
Current study	Gemcitabine	1,000	Day 1 cada 14	119	7	45	2.87	6.53	0
	Capecitabine	2,000	7 day cada 14						

*d* day, *RR* response rate, *mPFS* median progression-free survival, *mOS* median overall survival, *Neutrop* neutropenia

These results support the conclusion that under specific circumstances, including response to previous treatment lines; less aggressive tumor behavior; ECOG PS ≤2; and good liver, kidney, and bone marrow function, a new chemotherapy regimen, such as the one used here, might be useful in heavily pretreated mCRC.

Furthermore, achieving clinical benefit or response had an impact on survival (OS and PFS) and was an independent predictive factor for these survival outcomes, as demonstrated by univariate and multivariate analyses. These results lead us to believe that the benefit obtained with this chemotherapy alternative remains stable. This series revealed a strong tendency toward higher survival rates and greater clinical benefit among patients who had not received biological agents in previous regimens, supporting the rebound effect paradigm or a subgroup with a worse prognosis. The first concept was demonstrated after the suspension of monoclonal antibodies use, as well as the favorable outcomes achieved when these treatments were administered and maintained [24].

**Fig. 3:**
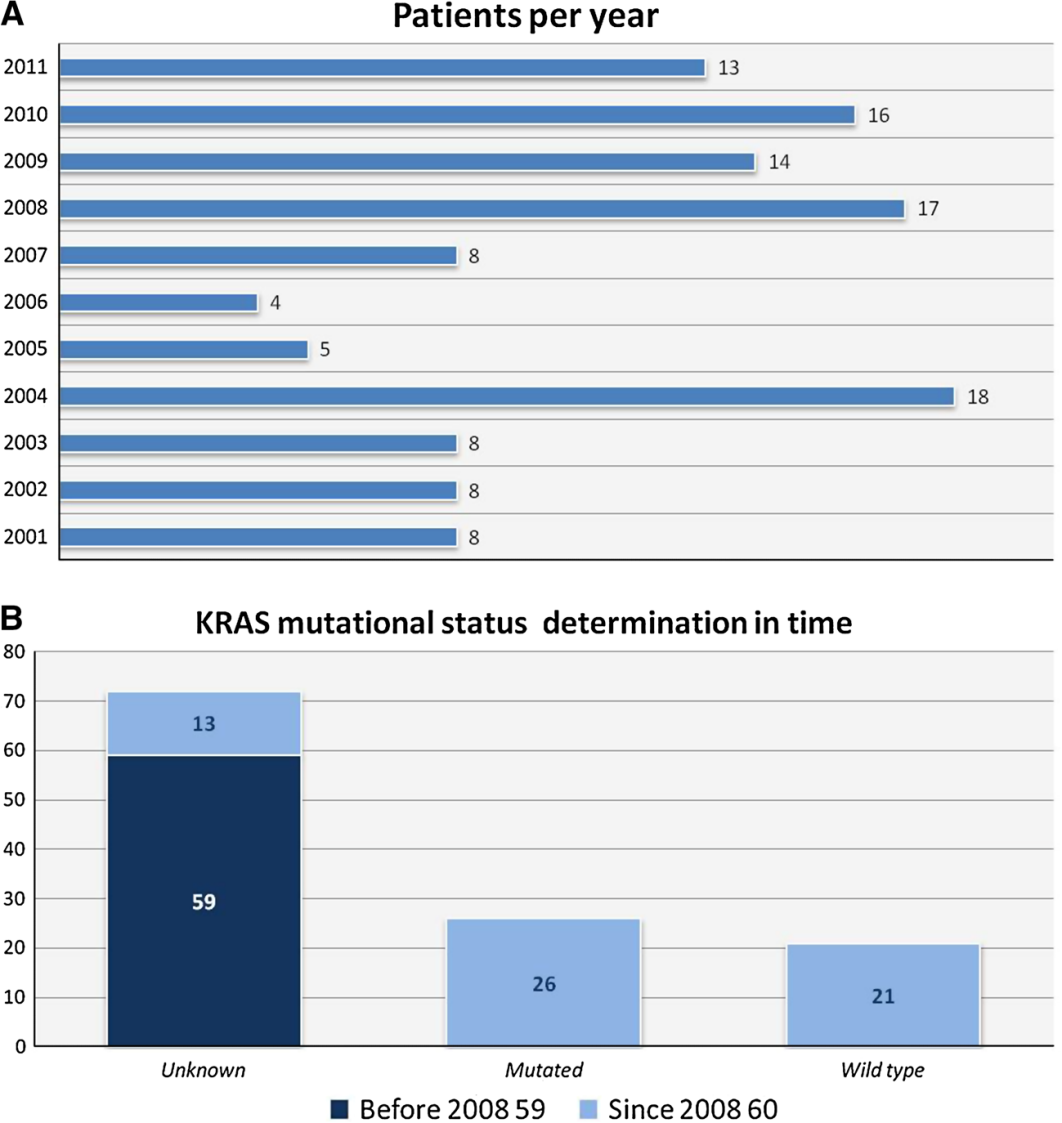
Number of patients per year (**a**) and K-ras mutation status before and since 2008 (**b**)

The major limitations of this study included the unicentric origins of the data, many years of data collection, the retrospective nature of the analysis, and the lack of a control group. As shown in Fig. 3, despite the long period of time, patients are distributed among the 10 years and following clinical guidelines, the state of the K-ras gene is known in most since 2008.

In summary, Gem–Cape seems to be active, possessing manageable toxicity and the capacity to accommodate heavily pretreated patients with mCRC who may not be eligible to participate in clinical trials. Nevertheless, further studies will improve our understanding of better chemotherapeutic approaches for “real-life” clinical practice.
